# Sex-Dependent Variations in Hypothalamic Fatty Acid Profile and Neuropeptides in Offspring Exposed to Maternal Obesity and High-Fat Diet

**DOI:** 10.3390/nu16030340

**Published:** 2024-01-24

**Authors:** Mayara da Nóbrega Baqueiro, Laís Angélica de Paula Simino, João Paulo Costa, Carolina Panzarin, Andressa Reginato, Marcio Alberto Torsoni, Letícia Ignácio-Souza, Marciane Milanski, Michael G. Ross, Kelly Pereira Coca, Mina Desai, Adriana Souza Torsoni

**Affiliations:** 1 Faculdade de Ciências Aplicadas, Universidade de Campinas, UNICAMP, Limeira 13484-350, São Paulo, Brazil; baqueiromayara@gmail.com (M.d.N.B.); nutricionistalais@gmail.com (L.A.d.P.S.); joaopcosta36@gmail.com (J.P.C.); carolinapanzarin@hotmail.com (C.P.); reginato.andressanutri@gmail.com (A.R.); torsoni@unicamp.br (M.A.T.); lmisouza@unicamp.br (L.I.-S.); marciane.milanski@fca.unicamp.br (M.M.); 2 Lundquist Institute at Harbor-UCLA Medical Center, Torrance, CA 90502, USA; mikeross@ucla.edu (M.G.R.); mdesai@lundquist.org (M.D.); 3 Department of Obstetrics and Gynecology, David Geffen School of Medicine, University of California Los Angeles at Harbor-UCLA, Torrance, CA 90502, USA; 4 Ana Abrao Breastfeeding Center, Escola Paulista de Enfermagem, Universidade Federal São Paulo, UNIFESP, São Paulo 04037-001, São Paulo, Brazil; kcoca@unifesp.br

**Keywords:** fatty acids, high-fat diet, hyperphagia, hypothalamus, metabolic programming

## Abstract

Maternal obesity and/or high-fat diet (HF) consumption can disrupt appetite regulation in their offspring, contributing to transgenerational obesity and metabolic diseases. As fatty acids (FAs) play a role in appetite regulation, we investigated the maternal and fetal levels of FAs as potential contributors to programmed hyperphagia observed in the offspring of obese dams. Female mice were fed either a control diet (CT) or HF prior to mating, and fetal and maternal blood and tissues were collected at 19 days of gestation. Elevated levels of linoleic acid were observed in the serum of HF dams as well as in the serum of their fetuses. An increased concentration of eicosadienoic acid was also detected in the hypothalamus of female HF-O fetuses. HF-O male fetuses showed increased hypothalamic neuropeptide Y (Npy) gene expression, while HF-O female fetuses showed decreased hypothalamic pro-opiomelanocortin (POMC) protein content. Both male and female fetuses exhibited reduced hypothalamic neurogenin 3 (NGN-3) gene expression. In vitro experiments confirmed that LA contributed to the decreased gene expression of Pomc and Ngn-3 in neuronal cells. During lactation, HF female offspring consumed more milk and had a higher body weight compared to CT. In summary, this study demonstrated that exposure to HF prior to and during gestation alters the FA composition in maternal serum and fetal serum and hypothalamus, particularly increasing *n*-6, which may play a role in the switch from POMC to NPY neurons, leading to increased weight gain in the offspring during lactation.

## 1. Introduction

The global obesity epidemic in the adult population has been extensively studied [1,2]. However, the alarming rates of obesity in women and children have become a major concern within the scientific community [3]. A Brazilian cohort study conducted in 2015 revealed that 47% of women were classified as overweight or obese before pregnancy [4]. In addition, obesity in children and adolescents (5–19 years old) has increased significantly. In 1975, the global prevalence of obesity was 0.7% among girls and 0.9% among boys. In 2016, these numbers increased to 5.6% and 7.8%, respectively [1].

Pregnancy and lactation are widely recognized as critical windows for perinatal programming, since adverse conditions during these periods have potential long-term metabolic consequences in adulthood [5,6]. Numerous studies have demonstrated that the consumption of a hyperlipidemic or hypercaloric diet, as well as maternal obesity, is strongly linked to negative outcomes in offspring. These outcomes include impaired glucose metabolism and liver function, hyperactivity, cardiovascular disease, and obesity [7,8,9,10,11,12,13,14]. Maternal obesity can affect weight gain in offspring through various mechanisms, including its impact on hyperphagic mechanisms [15,16].

The regulation of eating behavior involves a complex interplay of physiological and central signals from the hypothalamus, brainstem, and endocannabinoid system, as well as peripheral signals from the gastrointestinal tract and adipose tissue. In particular, the arcuate nucleus (ARC) within the hypothalamus plays a crucial role in appetite control, with specific anorexigenic and orexigenic neuronal populations Pro-opiomelanocortin (POMC)/cocaine- and amphetamine-regulated transcript (CART) and agouti-related peptide (AgRP)/neuropeptide Y (NPY), respectively, responding to these signals [17,18,19].

Optimal development of hypothalamic neurons during fetal development is essential for effective appetite regulation, and key regulators in this process include helix-loop-helix (bHLH) transcription factors such as hairy and enhancer of split-1 (Hes1), hairy and enhancer of split-5 (Hes5), Neurogenin 3 (Ngn3), and Achaete-scute homolog 1 (Mash1) [16,20]. Hes1 maintains the proliferation of neuroprogenitor cells (NPCs), while Mash1 and Ngn3 promote their differentiation into neurons [16,20]. Loss of function in Mash1 can lead to disrupted neurogenesis and a subsequent reduction in all neuronal lineages [21]. Additionally, Ngn3 plays a role in promoting the development of POMC neurons and inhibiting NPY neurons in the ARC [22]. Given that Pomc and Npy have opposing roles in regulating food intake, the interplay of Hes1, Mash1, and Ngn3 is crucial in maintaining energy homeostasis in mammals [16].

Previously, we demonstrated that offspring from obese dams fed a high-fat diet (HF-O) exhibited elevated levels of Npy and Agrp expression, along with a decrease in Ngn3, Mash1, and Pomc expression compared to offspring from control dams. These alterations in gene expression patterns favored hyperphagia and resulted in greater weight gain during postnatal life [23,24].

Considering the association between maternal obesity, lipotoxicity, and inflammation [25,26], one possible explanation for the hypothalamic alterations leading to hyperphagia in offspring of dams fed a HF-diet is the increased transfer of maternal saturated fatty acids (SFAs) across the placenta to the fetuses [27,28,29]. In a study on obese pregnant women, elevated levels of the SFA palmitate were detected in both maternal blood and the umbilical cord [30]. Excessive exposure to SFAs has been shown to cause dysfunction in the hypothalamus [31,32,33]. Additionally, experiments using NPCs from rodents demonstrated that treatment with palmitate inhibited neurogenesis [24,34]. SFAs are also known to impair mitochondrial function [35,36], which could in turn affect neuronal function and development [37,38].

On the other hand, the imbalance in the polyunsaturated fatty acids (PUFAs) *n*-6 (omega 6) and *n*-3 (omega 3) intake, characterized by increased consumption of *n*-6 and reduced consumption of *n*-3, has been associated with the development of leptin resistance and obesity [39]. Cheng et al. demonstrated that central administration of the *n*-6 PUFA arachidonic acid (ARA) promoted resistance to leptin signaling in the central nervous system and increased hypothalamic TNFa levels [40]. Thus, both alterations in saturated and unsaturated FAs may impact fetal hypothalamic development.

The aim of the present study was to investigate the effects of maternal HF diet consumption on fetal serum and hypothalamic fatty acid composition and explore its influence on the development of hyperphagia in the offspring.

## 2. Materials and Methods

### 2.1. Ethical Approval

The experiments were approved by the Ethical Committee for Animal Use (protocol no. 5639-1/2020) from the State University of Campinas (UNICAMP-Campinas, São Paulo, Brazil). The study was carried out in compliance with the guidelines and ethical standards for the care and use of laboratory animals of the “Brazilian Society of Science in Laboratory Animals” (SBCAL) and “Brazilian College of Animal Experimentation” (COBEA).

### 2.2. Animal Study

Five-week-old C57BL/6J females and mice were obtained from the Animal Breeding Centre at the University of Campinas (CEMIB) and maintained at 22 ± 1 °C and 12 h light/dark cycles with access to food and water ad libitum. Females were randomly assigned to a control diet (CT, 10% Kcal fat, 3.82 kcal/g; Research Diet D12450B, New Brunswick, NJ, USA) or to a HF diet (HF, 45% Kcal fat, 4.7 kcal/g; Research Diet D12451, New Brunswick, NJ, USA) (Table 1, Supplementary Tables S1 and S2). The fatty acid (FA) profile from the HF diet is presented in Supplementary Figure S1c,d. After an adaptation period of approximately 11 weeks, females were mated by housing two females with one male and maintained the same diet through pregnancy and lactation. Studies were undertaken on dams, fetuses, and offspring. Dams: Throughout the study period, weekly food intake and body weight were recorded. Following 8 weeks on the respective diets, fasting (12 h overnight fasting) blood was collected via the tail vein. On gestational day e19, CT (*n* = 27) and HF (*n* = 23) pregnant females were euthanized, and blood was collected and analyzed. The maternal adipose tissue (epigonadal, EPI; retroperitoneal, RET) was weighed to determine adiposity (adipose tissue weight/body weight × 100). Fetuses: At e19, fetuses and placentas were retrieved. Fetal body weight, length, placental weight, and placental efficiency (fetal weight/placental weight ratio) were measured. Subsequently, fetuses were decapitated, and blood and brains were collected. The hypothalamus was dissected for molecular analysis. Offspring: CT (*n* = 10) and HF (*n* = 10) pregnant females were allowed to deliver spontaneously, and after birth, pup body weight (D0) from CT-O and HF-O was recorded. Litter was standardized to 6 pups per dam (3 males and 3 females), and every 4 days milk intake of the pups was determined. Milk intake was performed using the “weight-suckle-weight regimen” [41]. Pups were separated from dams for 4 h, weighed and returned to dams for milk intake for 40 min, and then weighed again. Milk intake was calculated by subtracting post-intake body weight from post-fasting weight.

### 2.3. Serum Analysis

Serum insulin (Mouse Insulin Elisa Kit Invitrogen #KAQ1251), glucose (K082, Bioclin, Belo Horizonte, MG, Brazil), triglycerides (K117, Bioclin), and nonesterified fatty acids (NEFAs) (1138317500, Roche Diagnostics, Rotkreuz, Switzerland) assays were performed by enzymatic immunoassay.

### 2.4. Fetal Sexing

Fetal tails were collected at e19. The Extract-N-Amp Tissue PCR Kit (XNAT2R-1KT, Sigma-Aldrich, Saint Louis, MO, USA) was used to extract the fetal DNA. Conventional PCR was performed using the forward primer 5′-GAT GAT TTG AGT GGA AAT GTG AGG GTA-3′ and reverse primer 5-′CTT ATG TTT ATA GGC ATG CAC CAT GTA-3′ for amplification of the Xlr gene (Intron 6) from the X chromosome. The product was subjected to electrophoresis on a 3% agarose gel. Samples with two bands in the gel at 500 and 700 bp were considered female (XX), and samples with a band at 300 bp were considered male (XY).

### 2.5. Quantitative Real-Time PCR (qPCR)

Two fetal hypothalami from the same litter were used for RNA extraction using RNAzol RT (MRC, Cincinnati, OH, USA) in accordance with the manufacturer’s guidelines.

Nanodrop ND-2000 was used to quantify the RNA. Reverse transcription was performed with 3 µg of total RNA using the High Capacity cDNA Reverse Transcription kit (Applied Biosystems, Waltham, MA, USA). Relative gene expression was determined using the Taqman and Sybr green detection systems. Each real-time polymerase chain reaction (PCR) was performed with 20 ng/µL of complementary DNA on the ABI Prism 7500 Fast platform. The Taqman primers used for the target genes were: Hes1 (Mm01342805_m1); Hes5 (Mm00439311_g1); Ascl1 (Mm04207567_g1); Ngn3 (Mm00437606_s1); Pomc (Mm00435875_m1); Npy (Mm01410146_m1); Cart (Mm04210469_m1). Actb (4351315) was used as the housekeeping gene. The sequence of the Sybr green primer were: Pomc (F: CCC GCC CAA GGA CAA GCG TT, R: CTG GCC CTT CTT GTG CGC GT). Actb (F: GGA CTT CGA GCA AGA GAT GG. R: AGC ACT GTG TTG GCG TAC AG). The relative values determined by the comparative threshold cycle (Ct) method (2^−ΔΔCt^) were used to express the data. The sequence detection system 2.0.5 was used to analyze the data.

### 2.6. Chromatography Coupled to Mass Spectrometry (GC-MS)

Lipid was extracted from serum samples using methods described by Shirai et al. [42]. Hypothalamic lipids and diet lipids were similarly extracted but were modified for solid samples on a cold platform. Chromatography analysis was performed using GC MS-QP2010 (Shimadzu, Tokyo, Japan), Stabilwax column (30 m × 0.25 mm, and 0.25 μm of internal diameter; Restek, Bellefonte, PA USA), and ultrapure helium running gas. One milliliter of sample was injected using the automatic injector (AOC-20i). Briefly, the injector temperature was 250 °C with a gradual increase of 80 °C following 5 °C/min until 175 °C, and 3 °C/min until 230 °C, maintained for 20 min. The ionization occurred at a temperature of 200 °C and voltage of 70 eV, maintaining full scanning mode with an amplitude between 35 and 500 *m*/*z*, and 0.2 s by scanning.

### 2.7. Western Blot

Two fetal hypothalami from the same litter were used. The male and female samples were homogenized in ice-cold buffer (0.1 mol/L Tris, pH 7.4, 1% *v*/*v* Triton X-100 Thermo Fisher Scientific, Waltham, MA, USA 0.01mol/L EDTA, 0.1 mol/L sodium pyrophosphate, 0.1 mol/L sodium fluoride, 0.01 mg/mL aprotinin, 0.01 mol/L sodium vanadate, and 0.002 mol/L PMSF). The Bradford dye-binding method was employed to quantify protein levels. Then, 30 µg of protein was separated on a 15% SDS-PAGE gel and transferred to the nitrocellulose membrane. After blocking in 5% BSA in tris-buffered saline with tween (TBS-T), the membrane was incubated overnight at 4 °C in primary antibodies to NPY (sc-133080 1:500, Santa Cruz Biotechnology, Santa Cruz, CA, USA), POMC (ab25427 1:500, Abcam, Cambridge, MA, USA and ab_32893 1:500 Abcam), AGRP (sc_517457 1:500 Santa Cruz Biotechnology), NGN-3 (bs0922r 1:1000, Bioss and ab93488 1:2000, Abcam), MASH-1 (ab74065, 1:1000 Abcam). GAPDH (sc-25778, 1:1000 Santa Cruz Biotechnology) or βACTIN (ab8227, 1:1000 Abcam) were used as an endogenous control. Thereafter, the membranes were washed in TBS-T and incubated with the secondary antibodies for 90 min. Protein band visualization was detected by chemiluminescence using the chemiluminescence kit (34579, Super Signal West Pico Chemiluminescent Substrate, Thermo Fisher Scientific). The quantification of the intensity of the bands was performed using the Scion software (Scion Corp, MD, USA, version 1.0.0.1).

### 2.8. Immunofluorescence

Fetal brain was collected (e19) and stored in 4% PFA solution (paraformaldehyde) for 7 days, transferred to PBS 0.1M 10% sucrose solution with a gradual increase to PBS 30% solution overnight, frozen in OCT compound (Fisher HealthCare, Houston, TX, USA), and sectioned (15 µm coronal sections). Antigenic retrieval was performed on slices using Citrate-EDTA buffer for 40 min at 90 °C. Sections were blocked in 5% albumin (0.1M PBS + 0.25% Triton X-100) for 2 h at room temperature and incubated with primary antibodies overnight at 4 °C. The antibodies used were: NPY (1:100 #11976S, Cell Signaling© Danvers, MA, USA), POMC (1:50 ab254257, Abcam, Cambridge, MA, USA), and NEUN (1:500 ab104224, Abcam, Cambridge, MA, USA). Sections were incubated for 90 min with secondary antibodies Alexa 488 Rabbit (1:200, Invitrogen, Ref. Z25302), Alexa 568 mouse (1:200, Invitrogen, Ref. A11004). DAPI (1:1000 Life Technologies, Carlsbad, CA, USA) was used for nuclear labeling. Sections were visualized at 40X lens, captured using Upright LSM780-NLO Microscope (National Institute of Science and Technology on Photonics Applied to Cell Biology (INFABIC), University of Campinas), and quantified using ImageJ software (version 1.53 k, http://rsbweb.nih.gov/ij/ accessed on 11 August 2021). Cell counting was performed on 3 sections from the same fetus and 3 mice per group.

### 2.9. Cell Culture

For in vitro fatty acid treatment, mHypoA-POMC/GFP2 (RRID:CVCL_EP69) from murine hypothalamic neuron cell line (kindly donated by Dr. Denise Belsham, University of Toronto) was used. The cells were cultured in Low Glucose Dulbecco’s Modified Eagle’s Medium (DMEM), with 5% FBS and 1% penicillin-streptomycin solution (100 U/mL; 100 µg/mL, respectively) at 37 °C, 5% CO_2_, and 95% relative humidity. Cells were cultivated as monolayers between passages 22 and 24 in six-well plates approximately 24 h before experimentation. When cells were grown to 85% confluency, they were treated with linoleic acid (LA—100 µM) or vehicle (NaOH + BSA 3:1), as a control, for 24 h. Cells were then harvested for qPCR analysis.

### 2.10. Statistical Analysis

The software GraphPad Prism (Version 10.0.0, GraphPad Software Inc., San Diego, CA, USA) was used for data analysis. To determine outliers, the ROUT test was applied. A normality test was undertaken (Shapiro–Wilk). Student *t*-test or Mann–Whitney test was performed to compare between two groups. To evaluate two category variables, a two-way ANOVA test was performed, followed by the post-hoc Bonferroni test. If applicable, Pearson or Spearman correlation was applied. The alpha error was set at 5% (*p* ≤ 0.05) and data are presented as mean ± standard deviation (SD).

## 3. Results

### 3.1. Prepregnancy and Pregnancy HF Diet Consumption Promotes Increased Maternal Adiposity and Alters Serum Fatty Acid Profile

**Figure 1:**
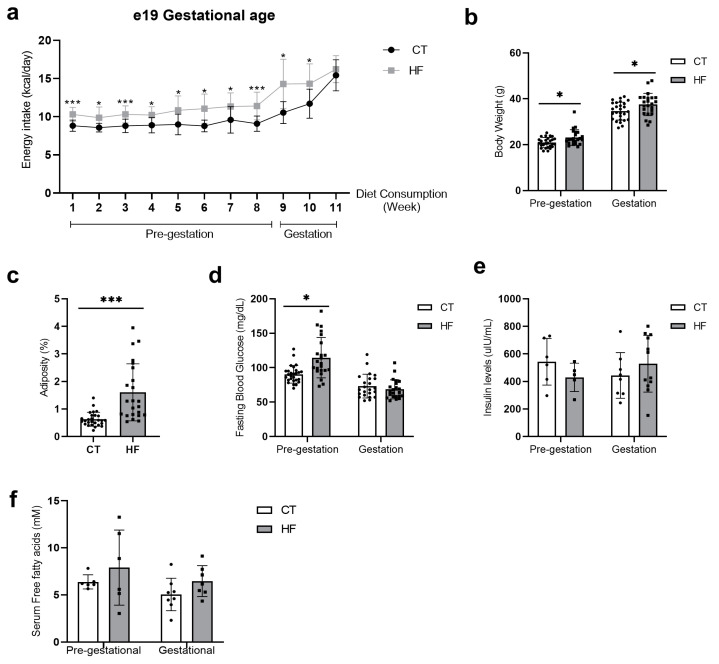
Metabolic profile assessment of high-fat (HF) and control (CT) dams at prepregnancy (before mating) and gestation (e19). (**a**) Energy intake (kcal/day) (CT *n* = 27 and HF *n* = 23). (**b**) Body weight (g) during prepregnancy and gestation (CT *n* = 27 and HF *n* = 22–23). (**c**) Adiposity at gestation (CT *n* = 27 and HF *n* = 23). (**d**) Prepregnancy and gestation fasting blood glucose (CT *n* = 27 and HF *n* = 22–23). (**e**,**f**) Serum insulin and free fatty acids during prepregnancy and gestation (CT *n* = 5–8 and HF *n* = 6–12). Bars represent the mean ± standard deviation. Unpaired *t*-test, Mann–Whitney test or two-way ANOVA * *p* < 0.05, *** *p* < 0.001, CT vs. HF.

Over an 8-week prepregnancy period, HF female mice consumed significantly increased calories (Figure 1a), which were mainly from fat and protein sources (Supplementary Figure S2a–d). At mating, HF females were significantly heavier (Figure 1b) with increased levels of serum glucose, though with similar levels of insulin and total free fatty acids as CT mice (Figure 1d–f). Throughout pregnancy, HF dams continued to consume significantly more calories and had increased body weight as compared to CT dams (Figure 1a,b). At gestational age e19, HF dams continued to have significantly increased adiposity (Figure 1c), though with comparable levels of serum glucose, insulin, and total free fatty acids to CT dams (Figure 1d–f). 

**Figure 2:**
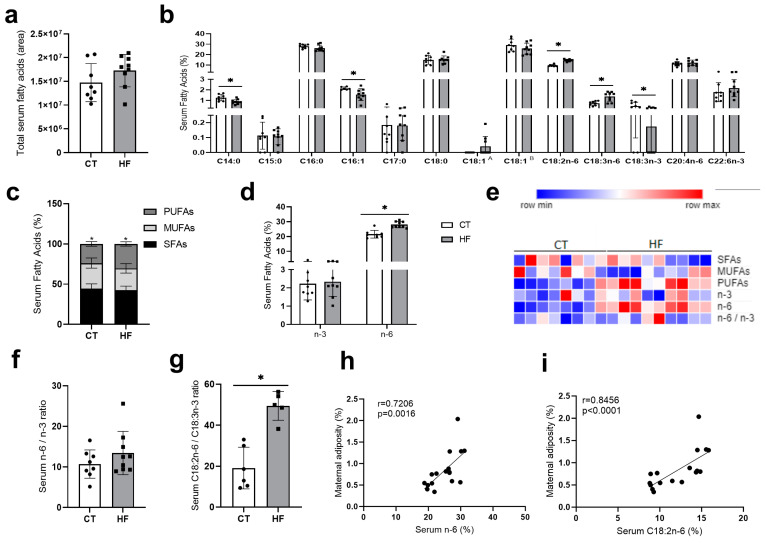
Maternal fatty acid profile assessment on the 19th day of gestation. (**a**) Total serum fatty acid area. (**b**) Serum fatty acids (%): methyl tetradecanoate (C14:0), pentadecanoic acid, methyl ester (C15:0), hexadecanoic acid, methyl ester (C16:0), 9-hexadecenoic acid, methyl ester (C16:1), heptadecanoic acid, methyl ester (C17:0), methyl stearate (C18:0), 6-octadecenoic acid, methyl ester (C18:1 A), 9-octadecenoic acid, methyl ester (C18:1 B), 9,12-octadecadienoic acid, methyl ester omega 6 (*n*-6) (C18:2*n*-6), gamma. Linolenic acid, methyl ester *n*-6 (C18:3*n*-6), 9,12,15-octadecatrienoic acid, methyl ester *n*-3 (C18:3*n*-3), 5,8,11,14-eicosatetraenoic acid, methyl ester *n*-6 (C20:4*n*-6), 4,7,10,13,16,19-docosahexaenoic acid, methyl ester *n*-3 (C22:6*n*-3). (**c**) Serum saturated fatty acids (SFAs), monounsaturated fatty acids (MUFAs), polyunsaturated fatty acids (PUFAs) proportion (%). (**d**) Serum fatty acids (%): *n*-3 (omega 3) and *n*-6 (omega 6). (**e**) Heat map of serum SFAs, MUFAs, PUFAs, *n*-3, and *n*-6. (**f**) Serum ratio: *n*-6/*n*-3. (**g**) Serum ratio: C18:2*n*-6/C18:3*n*-3. (**h**,**i**) Correlation between gestational maternal adiposity and maternal serum proportion of omega 6 and C18:2*n*-6. Unpaired *t*-test or Mann–Whitney test * *p* < 0.05 CT vs. HF. Spearman or Pearson test (* *p* < 0.05) CT *n* = 6–8, HF *n* = 5–9.

Although the proportion of total SFAs and monounsaturated fatty acids (MUFAs) was similar in HF and control dams, the proportion of specific fatty acids was altered. HF dams had an increased proportion of *n*-6-fatty acids linolenic acid (LA, C18:2*n*-6) and gamma-linolenic acid (C18:3*n*-6) and a decreased proportion of *n*-3-fatty acids alpha linolenic acid (ALA, C18:3*n*-3) resulting in an increased C18:2n6/C18:3n3 ratio as compared to CT dams (Figure 2). Additionally, there was a reduction in the proportion of C14:0 (saturated myristic acid) and C16:1 (monounsaturated palmitoleic acid) as compared to CT mice (Figure 2b). Notably, maternal adiposity was positively correlated with serum *n*-6 and linoleic acid (LA, C18:2*n*-6) (Figure 2h,i).

### 3.2. Sex-Specific Alteration in Serum Fatty Acid Profile in Fetuses of the Maternal HF Diet

**Figure 3:**
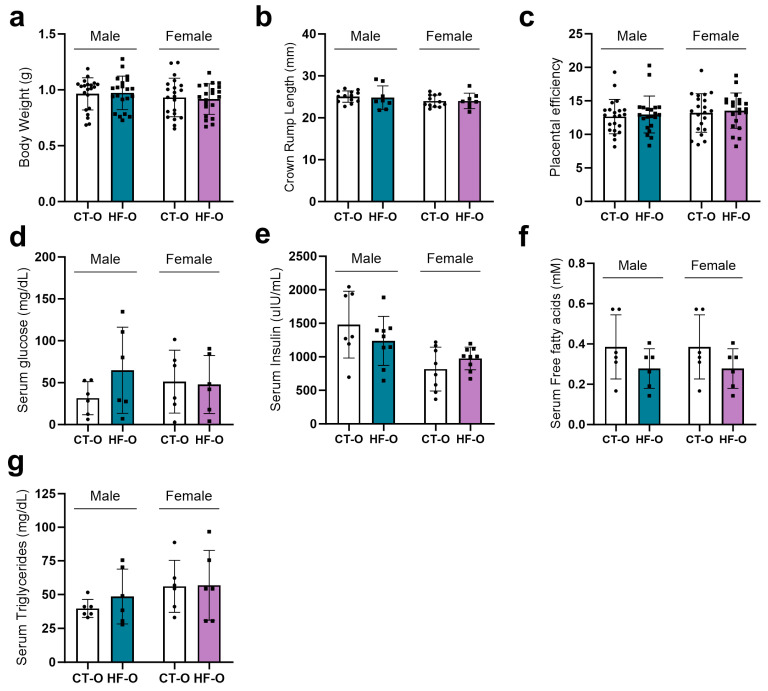
Metabolic data of control (CT-O) and high-fat (HF-O) fetuses. (**a**) Body weight (g) (CT-O *n* = 21 and HF-O-O *n* = 21). (**b**) Crown-rump length (mm) (CT-O *n* = 12–13 and HF-O *n* = 8). (**c**) Placental efficiency (CT-O *n* = 21 and HF-O *n* = 21–22). (**d**) Serum glucose, (**e**) insulin, (**f**) free fatty acids, and (**g**) triglycerides of male and female fetuses (CT-O = 6–7 and HF-O = 6–9). Bars represent the mean ± standard deviation.

At embryonic day 19 (e19), male and female fetuses from HF and CT dams showed similar body weight, crown-rump length, placental efficiency, and serum glucose, insulin, free fatty acids, and triglycerides (Figure 3).

**Figure 4:**
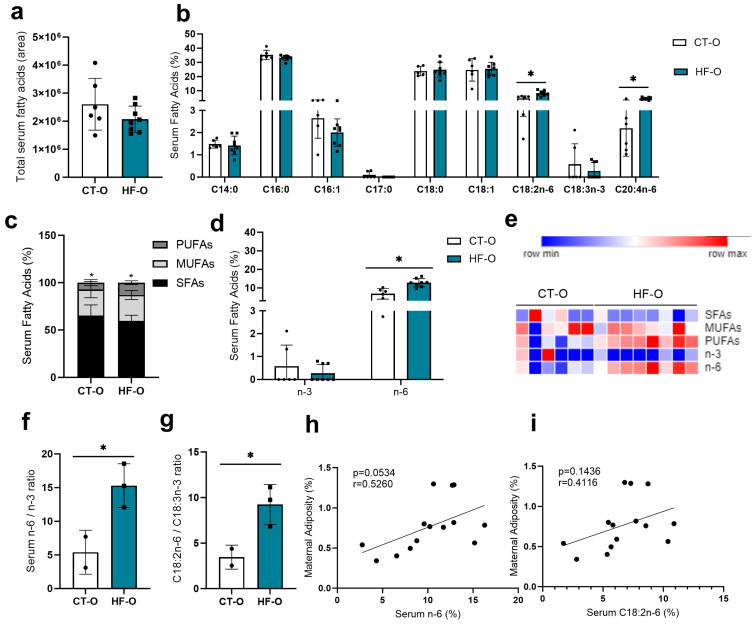
Serum fatty acid profile in male fetuses. (**a**) Total fatty acid serum (area) (CT-O *n* = 6, HF-O *n* = 8). (**b**) Relative amount of serum fatty acid (%): methyl tetradecanoate (C14:0), hexadecanoic acid methyl ester (C16:0), 9-hexadecenoic acid methyl ester, (Z)-(C16:1), heptadecanoic acid methyl ester (C17:0), methyl stearate (C18:0), 9-octadecenoic acid methyl ester (C18:1), 9,12-octadecadienoic acid methyl ester *n*-6 (C18:2*n*-6), 9,12,15-octadecatrienoic acid methyl ester (C18:3*n*-3), 5,8,11,14-eicosatetraenoic acid, methyl ester (C20:4*n*-6) (CT-O *n* = 5–6, HF *n* = 6–8). (**c**) Serum saturated fatty acids (SFAs), monounsaturated fatty acids (MUFAs), polyunsaturated fatty acids (PUFAs) proportion (%) (CT-O *n* = 6, HF-O *n* = 8). (**d**) Proportion (%) of serum *n*-3 (omega 3) and *n*-6 (omega 6) (CT-O *n* = 6, HF-O *n* = 8). (**e**) Heat map of serum SFAs, MUFAs, PUFAs, *n*-3, and *n*-6 (CT-O *n* = 6, HF-O *n* = 8. (**f**) Ratio of serum *n*-6 to *n*-3 (CT-O *n* = 2, HF-O *n* = 3). (**g**) Ratio of serum C18:2*n*-6–C18:3*n*-3 (CT-O *n* = 2, HF-O *n* = 3) (**h**,**i**). Correlation between gestational maternal adiposity and fetal serum proportions of *n*-6 and C18:2*n*-6. Unpaired *t*-test or Mann–Whitney test * *p* < 0.05, CT-O vs. HF-O. Spearman or Pearson test (* *p* < 0.05).

**Figure 5:**
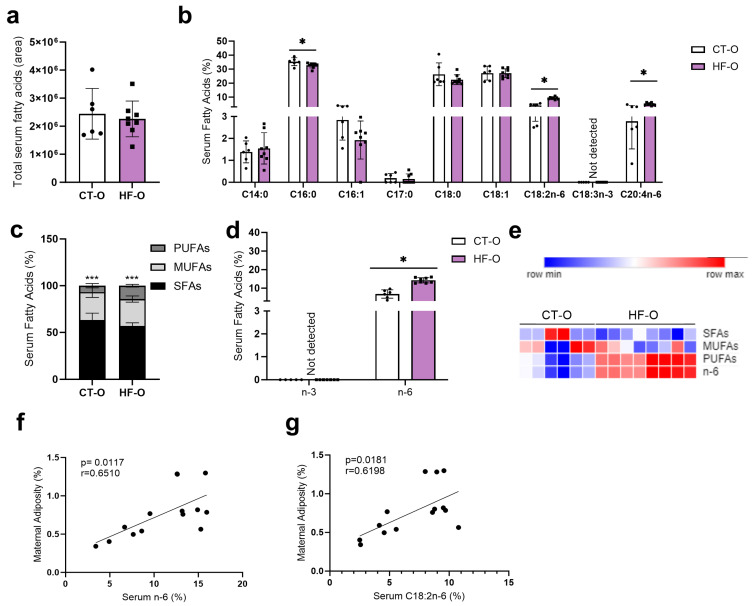
Serum fatty acid profile in female fetuses. (**a**) Total fatty acid serum (area) (CT-O *n* = 6, HF-O *n* = 8). (**b**) Relative amount of serum fatty acid (%): methyl tetradecanoate (C14:0), hexadecanoic acid methyl ester (C16:0), 9-hexadecenoic acid methyl ester, (Z)-(C16:1), heptadecanoic acid methyl ester (C17:0), methyl stearate (C18:0), 9-octadecenoic acid methyl ester (C18:1), 9,12-octadecadienoic acid methyl ester *n*-6 (C18:2*n*-6), 9.12, 15-octadecatrienoic acid methyl ester (C18:3*n*-3), 5,8,11,14-eicosatetraenoic acid, methyl ester (C20:4*n*-6) (CT-O *n* = 6, HF-O *n* = 8). (**c**) Serum saturated fatty acids (SFAs), monounsaturated fatty acids (MUFAs), and polyunsaturated fatty acids (PUFAs) proportion (%) (CT-O *n* = 6, HF-O *n* = 8). (**d**) Proportion (%) of serum *n*-3 (omega 3) and *n*-6 (omega 6) (CT-O *n* = 6, HF-O *n* = 8). (**e**) Heat map of serum SFAs, MUFAs, PUFAs, and *n*-6 (CT-O *n* = 6, HF-O *n* = 8). (**f**,**g**) Correlation between gestational maternal adiposity and fetal serum proportions of *n*-6 and C18:2*n*-6. Unpaired *t*-test or Mann–Whitney test * *p* < 0.05, CT-O vs. HF-O. Spearman or Pearson test (* *p* < 0.05, *** *p* < 0.0001).

Both male and female HF-O and CT-O fetuses had similar levels of serum fatty acid area (Figure 4a and Figure 5a) as well as similar proportions of SFAs, MUFAs, and *n*-3 fatty acids (Figure 4c–e and Figure 5c,d). However, the PUFAs and total *n*-6 were higher in HF-O as compared to the CT-O fetuses (Figure 4c,e and Figure 5c,e), with male and female fetuses having a higher proportion of C18:2*n*-6 and ARA (C20:4*n*-6) (Figure 4b and Figure 5b). Furthermore, only the HF-O male fetuses showed higher *n*-6/*n*-3 and C18:2*n*-6/C18:3*n*-3 ratios as compared to the CT-O fetuses (Figure 4f,g), with the female showing a decrease in saturated palmitic acid (C16:0) as compared to the CT-O fetuses (Figure 5b). In both HF-O male and female fetuses, levels of *n*-6 (Figure 4h and Figure 5f) were positively correlated with maternal adiposity. In female, but not in male fetuses (Figure 4i and Figure 5g), C18:2*n*-6 levels were positively correlated with maternal adiposity. 

### 3.3. Hypothalamic Fatty Acid C20:2n-6 Is Associated with Maternal Adiposity

**Figure 6:**
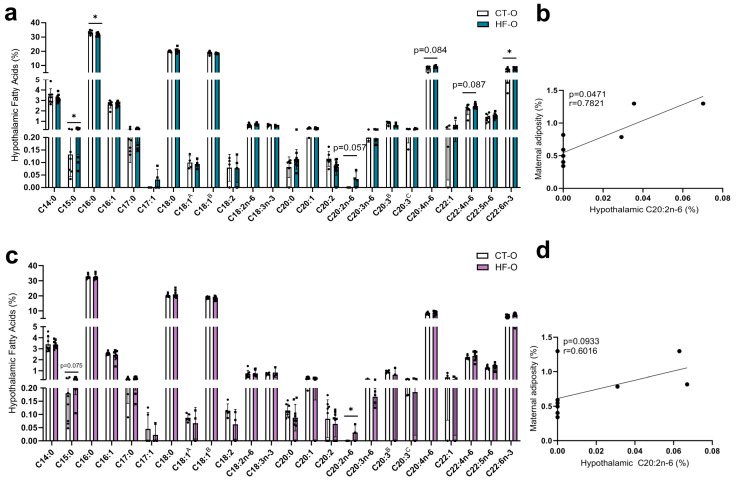
Fetal male and female hypothalamic fatty acid profiles. (**a**,**c**) Relative amount of male and female hypothalamic fatty acids (%): methyl tetradecanoate (C14:0), pentadecanoic acid, methyl ester (C15:0), hexadecanoic acid, methyl ester (C16:0), 9-hexadecenoic acid, methyl ester (C16:1), heptadecanoic acid, methyl ester (C17:0), cis-10-heptadecenoic acid, methyl ester (C17:1), methyl stearate (C18:0), 6-octadecenoic acid, methyl ester (C18:1A), 9-octadecenoic acid, methyl ester (C18:1 B), 6,9-octadecadienoic acid, methyl ester (C18:2), 9,12-octadecadienoic acid, methyl ester *n*-6 (C18:2*n*-6), 9,12,15-octadecatrienoic acid, methyl *n*-3 ester (C18:3*n*-3), eicosanoic acid, methyl ester (C20:0), cis-11-eicosenoic acid, methyl ester (C20:1), 8,11-eicosadienoic acid, methyl ester (C20:2), cis-11,14-eicosadienoic acid, methyl ester (n6) (C20:2*n*-6), 8,11,14-eicosatrienoic acid, methyl ester *n*-6 (C20:3*n*-6), cis-5,8,11-eicosatrienoic acid, methyl ester (C20:3B), 7,10,13-eicosatrienoic acid, methyl ester (C20:3C), 5,8,11,14-eicosatetraenoic acid, methyl ester *n*-6 (C20:4*n*-6), 13-docosenoic acid, methyl ester (C22:1), cis-7,10,13,16-docosatetraenoic acid, methyl ester *n*-6 (C22:4*n*-6), methyl 4,7,10,13,16-docosapentaenoate *n*-6 (C22:5*n*-6), 4,7,10,13,16,19-docosahexaenoic acid, methyl ester *n*-3 (C22:6*n*-3) (CT-O = 4–8 and HF-O = 4–10). (**b**) Correlation between gestational maternal adiposity and male hypothalamic proportion of C20:2*n*-6. (**d**) Correlation between gestational maternal adiposity and female hypothalamic proportion of C20:2*n*-6. Unpaired *t*-test or Mann–Whitney test * *p* < 0.05, CT-O vs. HF-O. Spearman or Pearson test (* *p* < 0.05).

At e19, the hypothalamic levels of PUFAs MUFAs, SFA, *n*-3, and *n*-6 were comparable in male and female HF-O and CT-O fetuses (Supplementary Figure S3). However, there were significant differences in the hypothalamic fatty acid profile. In HF-O male fetuses, pentadecanoic acid (C15:0), eicosadienoic acid (C20:2*n*-6), and docosahexaenoic acid (DHA, C22:6*n*-3) levels were increased, whereas in HF-O female fetuses, C20:2*n*-6 was increased as compared to CT-O (Figure 6a,c). Furthermore, it was found that maternal adiposity exhibited a positive correlation with the levels of hypothalamic C20:2*n*-6 in male fetuses (Figure 6b). 

### 3.4. Sex-Specific Changes in Expression of Hypothalamic Neuropeptides (NPY, POMC) and the bHLH Gene in HF-O Fetuses

**Figure 7:**
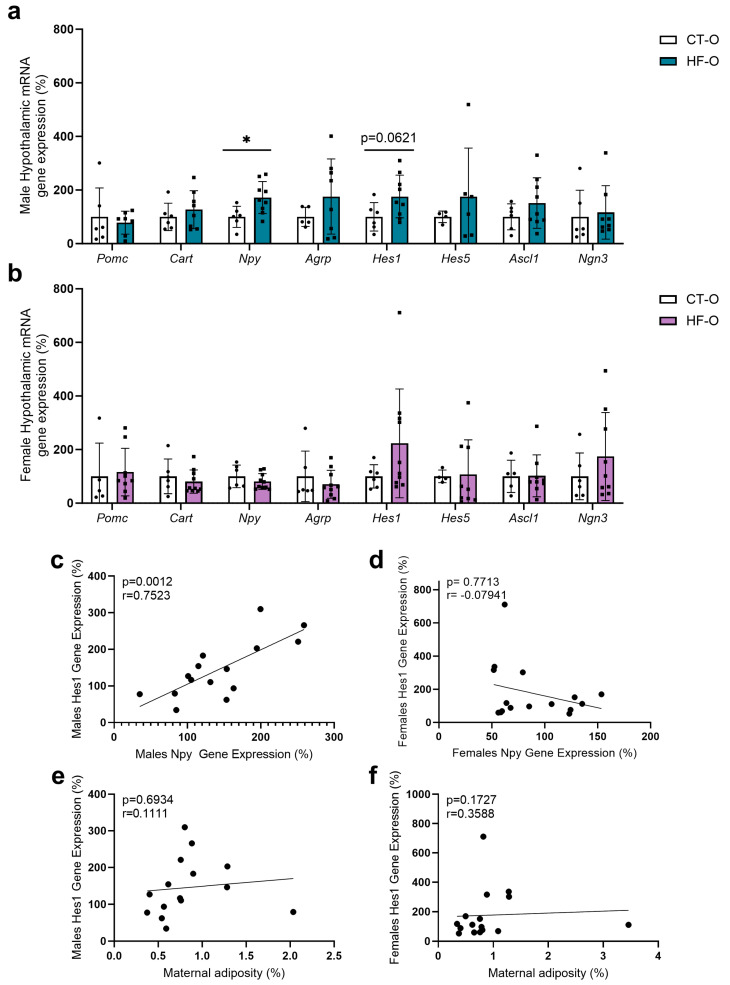
Hypothalamic gene expression is related to appetite control in male and female fetuses. Hypothalamic relative gene expression (q-RT-PCR) of *Pomc, Cart, Npy, Agrp, Hes1, Hes5, Ascl1, and Ngn3* of CT-O and HF-O (**a**) male and (**b**) female. Housekeeping gene: *Actb*. (*n* = 4–10/group). (**c**,**e**) Correlation between *Hes1* and *Npy* gene expression in males and females, respectively. (**d**,**f**) Correlation between maternal adiposity and *Hes1* gene expression in males and females, respectively. Unpaired *t*-test or Mann–Whitney test * *p* < 0.05, CT-O vs. HF-O. Spearman or Pearson test (* *p* < 0.05).

**Figure 8:**
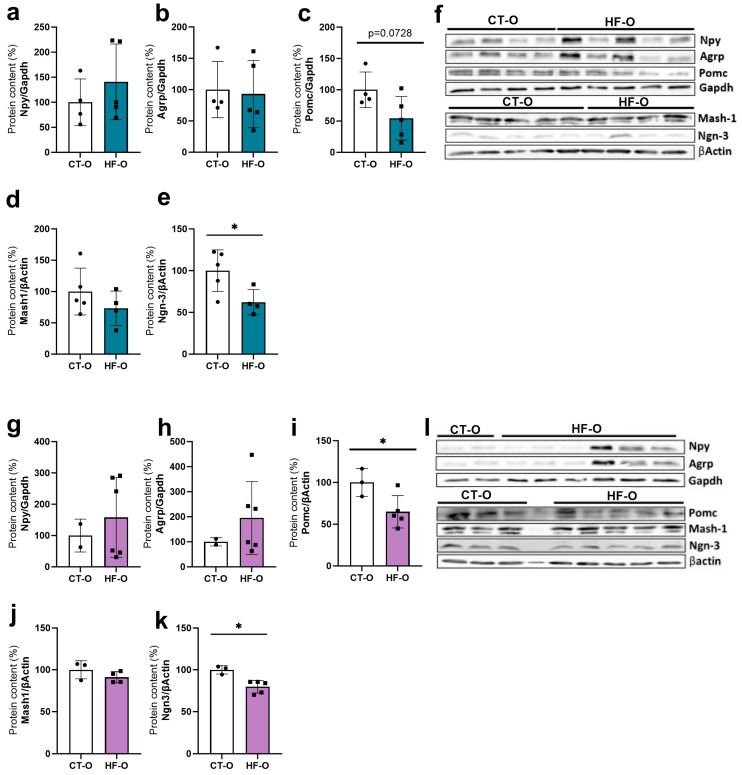
Hypothalamic protein content of proteins related to appetite control in male and female fetuses. Protein content (%) of NPY, AGRP, MASH-1, and NGN-3 of CT-O and HF-O (**a**–**f**) male and (**g**–**l**) female. (*n* = 2–5/group). Control: GAPDH or βACTIN. Unpaired *t*-test or Mann–Whitney test * *p* < 0.05, CT-O vs. HF-O.

**Figure 9:**
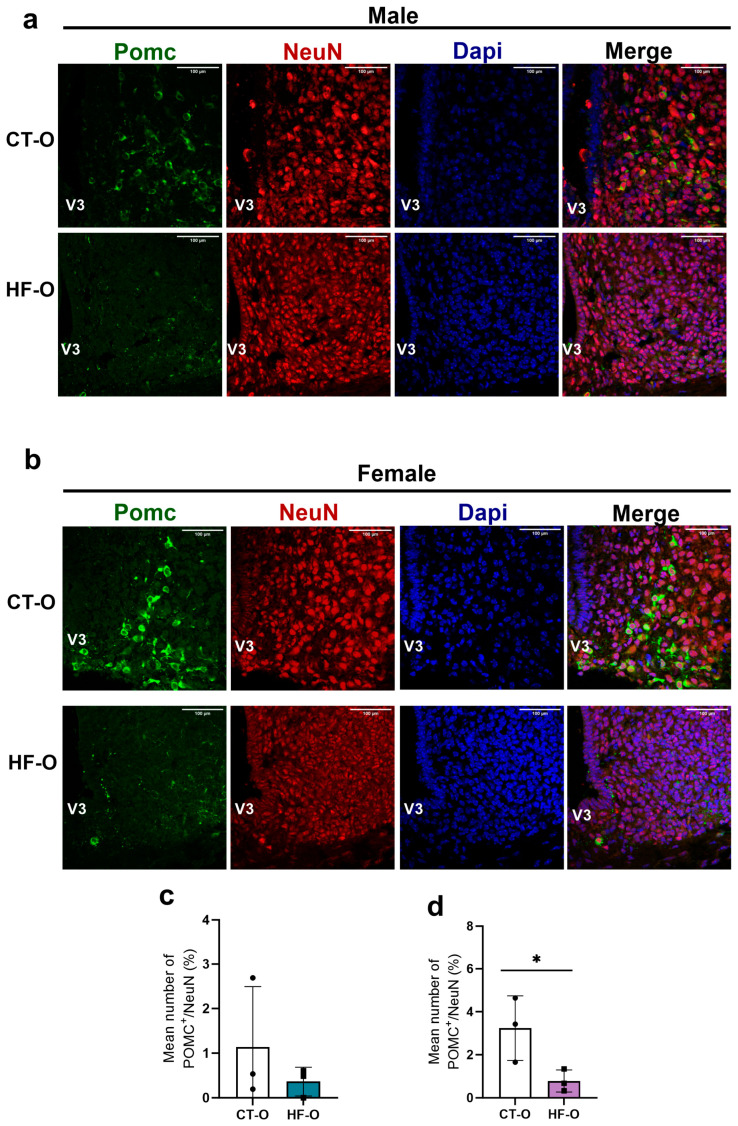
POMC content in ARC of male and female fetuses. Confocal microscopic images illustrating POMC^+^ cells (green), NeuN^+^ cells (red), and DAPI nuclear labeling (blue) were performed on coronal sections (15 µm) of the brain of (**a**) male and (**b**) female fetuses. (**c**,**d**) Stereology image analysis of POMC in the fetal hypothalamus. (*n* = 3/group). Unpaired *t*-test * *p* < 0.05, CT-O vs. HF-O.

**Figure 10:**
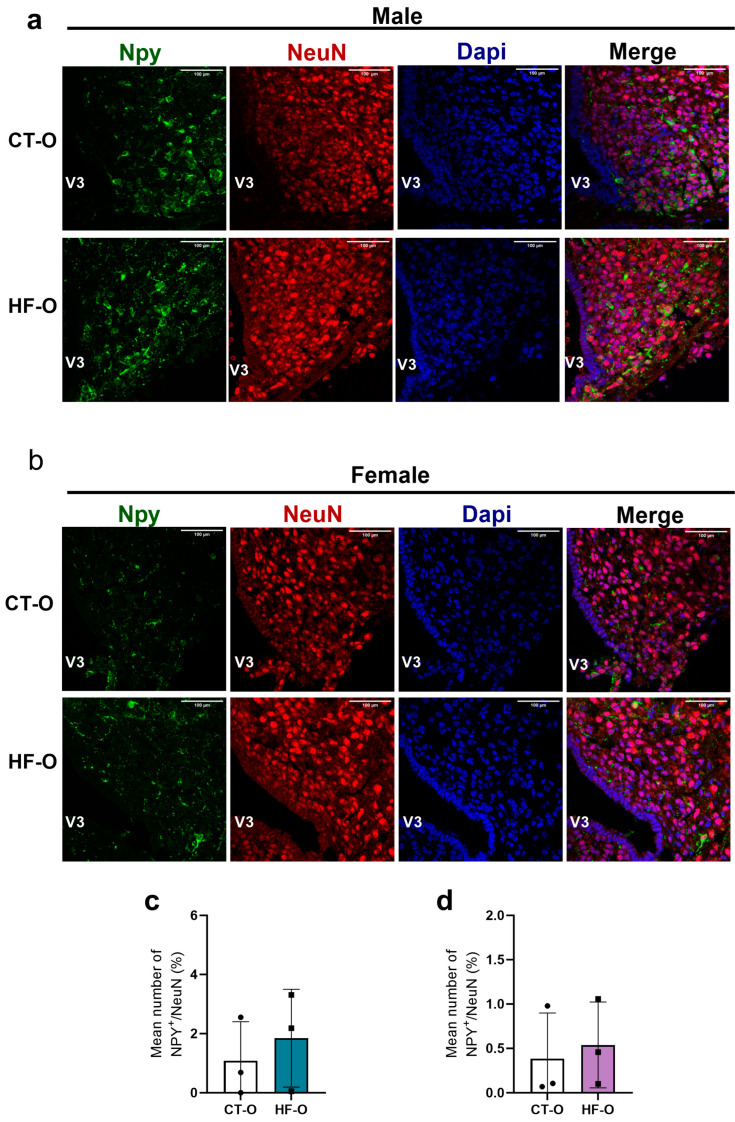
NPY content in ARC of male and female fetuses. Confocal microscopic images illustrating NPY^+^ cells (green), NeuN^+^ cells (red), and DAPI nuclear labeling (blue) were performed on coronal sections (15 µm) of the brain of (**a**) male and (**b**) female fetuses. (**c**,**d**) Stereology image analysis of NPY in fetal hypothalamus. (*n* = 3/group).

In HF-O male fetuses, hypothalamic Npy gene expression increased with a trend for increased Hes1 as compared to CT-O male fetuses (Figure 7a). However, the protein content of NPY and POMC was similar to that of CT-O male fetuses (Figure 8a–f). Conversely, in HF-O females, although no changes were noted in mRNA gene expression, the protein content of hypothalamic POMC and NGN3 was significantly reduced (Figure 8g–l). Stereology analysis confirmed a reduction in POMC labeling in neurons in the hypothalamus of HF-O females as compared to CT-O females (Figure 9b,d), but not in male fetuses (Figure 9a,c). No changes in NPY labeling were noted in neurons in both males and females (Figure 10). Correlation analysis showed a positive correlation between the hypothalamic expression of Npy and Hes1 in HF-O male fetuses (Figure 7c), but not between hypothalamic Hes1 expression and maternal adiposity (Figure 7e). No significant correlations were observed among female fetuses (Figure 7d,f).

### 3.5. Fetal Changes in the Expression of Hypothalamic Neuropeptides and bHLH Factors Impact Milk Intake and Increase Weight Gain in HF-O

**Figure 11:**
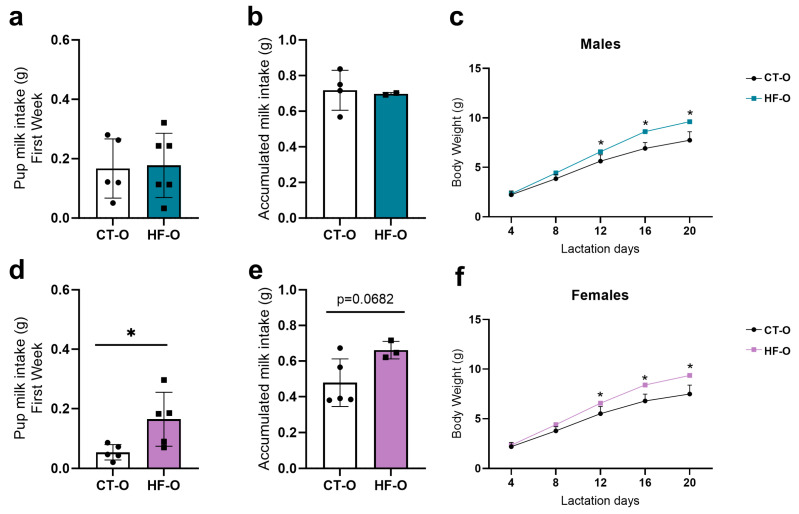
Milk intake and body weight in male and female offspring during the lactation period. (**a**,**d**) Male and female milk intake (g) at the first week of lactation (CT-O *n* = 5, HF-O *n* = 5–6). (**b**,**e**) Male and female accumulated milk intake (g) on days 4,8,12,16, and 20 of lactation (CT-O *n* = 4–5, HF-O *n* = 2–3). (**c**,**f**) Male and female body weight (g) at lactation. *n* = 10/group. Unpaired *t*-test * *p* < 0.05, CT-O vs. HF-O.

After birth, the milk intake of HF-O and CT-O male offspring was similar (Figure 11a,b). However, HF-O females displayed an increase in accumulated milk intake from days 4–8 of lactation (Figure 11d) and a trend towards increased milk intake on days 4, 8, 12, 16, and 20 (Figure 11e) as compared to CT-O females. Although there were no changes in milk consumption in male offspring, both male and female HF-O showed an increase in body weight from the 12th day of life (Figure 11c,f).

### 3.6. In Vitro Treatment of Hypothalamic Neuronal Cell Line with Linoleic Acid (LA) in Reduces Expression of Pomc and Ngn3 Genes

**Figure 12:**
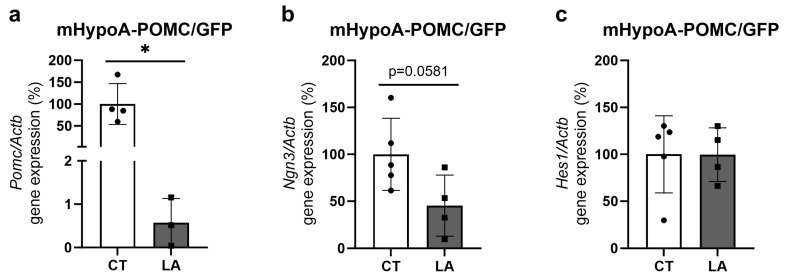
Gene expression of neuropeptides and bHLH factors in linoleic acid (LA)-treated hypothalamic neuronal cells. Relative expression (q-RT-PCR) of (**a**) *Pomc*, (**b**) *Ngn3*, and (**c**) *Hes1*. Housekeeping gene: *Actb.* (*n* = 3–6/group). Unpaired *t*-test * *p* < 0.05, CT vs. LA.

In an in vitro experiment using hypothalamic neuronal cells (mHypoA-POMC/GFP2) treated with linoleic acid (LA), we observed a decrease in *Pomc* gene expression and a trend towards a decrease in the bHLH factor *Ngn3*. However, there were no significant changes detected in the levels of *Hes1* transcripts following LA treatment (Figure 12a–c).

## 4. Discussion

This study reports that maternal HF diet consumption during pregnancy (1) alters specific fatty acid proportions, especially *n*-6, which is increased in maternal serum, fetal serum, and fetal hypothalamus, (2) causes a sex-dependent alteration in gene/protein expression of fetal hypothalamic appetite/satiety neuropeptide in a direction that promotes increased milk intake, and (3) changes the expression of bHLH genes associated with specific neuronal differentiation. Lastly, using hypothalamic neuronal cultures, we confirmed that in vitro treatment with *n*-6 (linoleic acid) modulates the expression of Pomc and Ngn3.

At mating, female mice fed a HF diet were obese, as evident by increased body weight and adiposity, higher energy intake, and elevated glycemia levels. It is widely established that the offspring of HF dams exhibit rapid body weight gain and increased adiposity, partly due to increased energy intake [6,14,23,43,44], suggesting a potential impairment in the development of neuronal circuits responsible for regulating energy homeostasis. Although the mechanisms have not been fully elucidated, the significance of long-chain polyunsaturated fatty acids (LC-PUFAs) in neuronal development has been the subject of investigation [45].

At the end of gestation, the HF dams and their fetuses exhibited an elevated serum C18:2*n*-6 (LA)/C18:3*n*-3 (ALA) ratio. The presence of LA, ALA, and other LC-PUFAs derivatives of essential fatty acids, such as ARA and DHA, in fetuses depends on the process of placental transport [46]. The HF diet provided to the females had a significantly higher *n*-6/*n*-3 ratio, which likely contributed to the observed increase in maternal and male and female fetal serum *n*-6 levels. Furthermore, the competition between fatty acids (*n*-6/*n*-3) for the same enzymes involved in their conversion into active forms, such as ARA, eicosapentaenoic acid (EPA), and DHA, is well-established [47]. Therefore, the composition of lipids in the diet can also contribute to changes in the maternal serum lipid profile, influenced by the hepatic conversion of ARA, EPA, and DHA. These findings are consistent with the study that demonstrated the impact of maternal consumption of a distinct HF diet on alterations in the fatty acid composition of both dams and offspring, resulting in elevated levels of *n*-6 fatty acids in milk and serum [48]. On the other hand, Haggarty and colleagues evaluating the lipid transport in the human placenta showed that there is a preferential transfer of *n*–3 fatty acids from the maternal to fetal circulation [49]. Accordingly, maternal HF diet consumption could disrupt the efficiency and selectivity of fatty acid transport through the placenta, as demonstrated by the Slc27a4 gene in rodent placenta [50], ultimately resulting in the alterations observed (higher *n*-6/*n*-3 ratio) to the fetal serum profile.

LC-PUFAs, particularly the *n*-3 fatty acids, play a vital role in the development of the brain, visual system, immune system, and metabolism [51]. In humans, gestation is a crucial period for the accumulation of *n*-3 and *n*-6 fatty acids in the fetal brain [52,53,54,55,56]. There is limited available data regarding the specific quantities of *n*-3 and *n*-6 fatty acids in different types of brain cells. However, DHA and ARA are the primary lipid species found in the cell membranes of both rodent and human brains [57,58]. Studies have shown that DHA and EPA can be found in glial cells and play an important role in neuroinflammation and myelin formation [59,60,61,62]. Although in the present study, specific brain cell types were not studied, we showed that, during the fetal period (e19), female and male fetuses exposed to a maternal HF diet also exhibited elevated levels of eicosadienoic acid (C20:2 *n*-6) fatty acids in the hypothalamus. The increase in LC-PUFAs coincided with a reduction in the hypothalamic protein content of NGN3, a protein associated with POMC neuron differentiation [22], in both HF-O females and males. Despite this, there were sex-specific changes in neuropeptide expression. HF-O female fetuses showed decreased POMC protein expression, whereas HF-O male fetuses showed increased NPY gene expression.

To assess the impact of these changes on appetite regulation, we measured milk consumption and body weight as endpoints. Although both female and male HF-O showed increased body weight, only females showed greater milk consumption. We can attribute the greater weight gain in HF-O females to the increased milk intake in this group, while the increase in body weight in HF-O males may be a result of more efficient conversion of food ingested during the lactation period or reduced energy expenditure. The elevated feed efficiency rate in male HF-O (CT = 8.22 ± 1.16 vs. HF = 10.88 ± 0.38, *p* = 0.04) represents the powerful cumulative efficiency with which male pups utilized dietary nutrients for maintenance and body mass gain (lean mass and lipid accretion) compared to the control group, thus promoting the conversion of ingested food into body mass. These results may suggest that the increase in feed efficiency in males was programmed by the maternal HF diet, the same observed by Vido and coworkers, who showed that maternal obesity was able to increase gross feed efficiency in adult offspring [63].

The increase in milk intake in HF-O females is consistent with reduced Pomc expression and satiety. In HF-O males, increased NPY is also consistent with increased body weight. The comparable milk intake in HF-O and control males may be due to the low number of animals studied or limitations in weight-suckle-weight measurements.

Neurogenesis relies significantly on fatty acid synthesis, especially during the neuronal differentiation process [64]. Additionally, in vitro analyses conducted on hypothalamic neurons confirmed that linoleic acid is capable of modulating the expression of Pomc and Ngn3. These findings suggest that elevated *n*-6 levels in the hypothalamus, even in the absence of increased levels of SFA, can impact the differentiation of POMC neurons, leading to hyperphagic behavior during lactation. Furthermore, Hejr et al. demonstrated that the exposition of NPCs to a high ratio of *n*-6 linoleic acid to *n*-3 linolenic acid (3:1) led to a reduction in the size and number of NPCs, suggesting the important role of the *n*-6/*n*-3 ratio in neuronal proliferation and differentiation processes [65].

Our results suggest that the elevated levels of *n*-6 fatty acids detected in the serum of HF dams and HF-O fetuses serum/hypothalamus may drive changes in neurodifferentiation in HF-O during development. In a previous study, using a similar model, we showed that offspring of obese dams exhibit an increase in hypothalamic neuropeptide Y (NPY)-positive neurons and higher body weight compared to control offspring, persisting into adulthood [16,23]. This suggests that alterations in neuronal differentiation might contribute to lasting hyperphagic behavior in offspring. Our laboratory previously demonstrated that offspring of HF dams had higher milk intake compared to offspring of control dams, even when considering other factors such as litter size [66,67]. Additionally, HF dams also exhibited increased milk production compared to control dams [66,67]. 

Collectively, our results enhance our understanding of the intricate interactions between exposure to a maternal HF diet and adverse outcomes in offspring, indicating that higher levels of *n*-6 fatty acids in the maternal diet and serum correlate with elevated *n*-6 levels in the serum and hypothalamus of offspring. This, in turn, exerts a significant effect on the NGN3 expression and neurodifferentiation of POMC neurons, resulting in greater hyperphagia and weight gain in descendants. 

## 5. Conclusions

Our findings indicate that the consumption of a HF diet during prepregnancy and pregnancy can potentially lead to detrimental alterations in the fatty acid profile and the expression of neuropeptides involved in central appetite control during the fetal period. These changes may contribute to the development of an obesogenic phenotype in offspring.

## Figures and Tables

**Table 1:** Control diet (Research Diet D12450B) and HF diet (Research Diet D12451) composition. Protein Sources: casein and L-cystine. Carbohydrates: sucrose, corn starch, and maltodextrin. Lipids: soybean oil and lard.

	Control Diet	HF Diet
Carbohydrates (%)	70	35
Proteins (%)	20	20
Lipids (%)	10	45
Energy Density (kcal/g)	3.82	4.7

## Data Availability

The original contributions presented in the study are included in the article/supplementary material. Further inquiries can be directed to the corresponding author.

## Supplementary Materials

The following supporting information can be downloaded at: https://www.mdpi.com/article/10.3390/nu16030340/s1, Table S1: Nutritional Composition. Control Diet Research Diet D12450B, New Brunswick, NJ, USA; Table S2: Nutritional Composition. High Fat Research Diet D12451, New Brunswick, NJ, USA; Figure S1: Diet fatty acids profile; Figure S2: Caloric contribution of macronutrient intake of females fed high fat (HF) or control (CT) diet; Figure S3: Fetal hypothalamic fatty acids profile.
