# Supplementary material for: Sex-Dependent Variations in Hypothalamic Fatty Acid Profile and Neuropeptides in Offspring Exposed to Maternal Obesity and High-Fat Diet
Source: Nutrients. 2024 Jan 24;16(3):340. doi: 10.3390/nu16030340 (PMC10857148; doi:10.3390/nu16030340)
Supplement: Supplementary file 1 [file nutrients-16-00340-s001.zip › nutrients-2771334-supplementary.pdf]

Supplementary

Table S1. Nutritional composition. Control Diet Research Diet D12450B, New Brunswick, NJ, USA.

Control Diet		
**Class description**	**Ingredients**	**Grams**
Protein	Casein, Lactic, 30 Mesh	200.00 g
Protein	Cystine, L	3.00 g
Carbohydrate	Sucrose, Fine Granulated	354.00 g
Carbohydrate	Starch, Corn	315.00 g
Carbohydrate	Lodex 10	35.00 g
Fiber	Solka Floc, FCC200	50.00 g
Fat	Soybean Oil, USP	25.00 g
Fat	Lard	20.00 g
Mineral	S10026B	50.00 g
Vitamin	Choline Bitartrate	2.00 g
Vitamin	V10001C	1.00 g
Dye	Dye, Yellow FD&C #5, Alum. Lake 35-42%	0.05 g
	**Total:**	1055.05 g

Table S2. Nutritional Composition. High Fat Research Diet D12451, New Brunswick, NJ, USA.

High Fat Diet		
**Class description**	**Ingredients**	**Grams**
Protein	Casein, Lactic, 30 Mesh	200.00 g
Protein	Cystine, L	3.00 g
Carbohydrate	Sucrose, Fine Granulated	176.80 g
Carbohydrate	Lodex 10	100.00 g
Carbohydrate	Starch, Corn	72.80 g
Fiber	Solka Floc, FCC200	50.00 g
Fat	Lard	177.50 g
Fat	Soybean Oil, USP	25.00 g
Mineral	S10026B	50.00 g
Vitamin	Choline Bitartrate	2.00 g
Vitamin	V10001C	1.00 g
Dye	Dye, Red FD&C #40, Alum. Lake 35-42%	0.05 g
	**Total:**	858.15 g

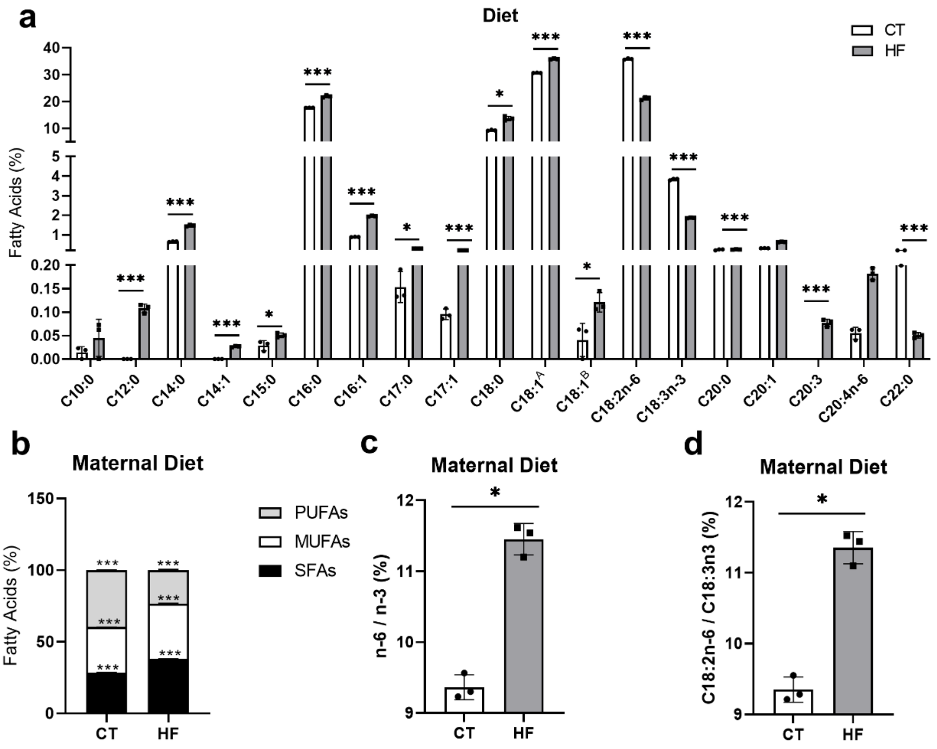

Figure S1. Diet fatty acids profile. (a) Relative amount of hypothalamic fatty acid (%):Decanoic acid, methyl ester (C10:0); Dodecanoic acid, methyl ester (C12:0); Methyl tetradecanoate (C14:0); Pentadecanoic acid, methyl ester (C15:0); Hexadecanoic acid, methyl ester (C16:0); 9-Hexadecenoic acid, methyl ester, (Z)- (C16:1); Heptadecanoic acid, methyl ester (C17:0); cis-10-Heptadecenoic acid, methyl ester (C17:1); Methyl stearate (C18:0); 9-Octadecenoic acid, methyl ester, (E)- (C18:1 A);11-Octadecenoic acid, methyl ester (Trans) (C18:1 B); 9,12-Octadecadienoic acid (Z,Z)-, methyl ester, n-6 (C18:2,n-6); 9,12,15-Octadecatrienoic acid, methyl ester, (Z,Z,Z)(n-3)(C18:3,n-3); Eicosanoic acid, methyl ester (C20:0); 11-Eicosenoic acid, methyl ester (C20:1); 7,10,13-Eicosatrienoic acid, methyl ester (C20:3); 5,8,11,14-Eicosatetraenoic acid, methyl ester, (all-Z), n-6 (C20:4n-6); Docosanoic acid, methyl ester (C22:0). (b) Hypothalamic Saturated Fatty Acids (SFAs), Monounsaturated Fatty Acids (MUFAs), Polyunsaturated Fatty Acids (PUFAs), (c) Ratio of omega 6 (n-6) to omega 3 (n-3) in maternal diet. (d) ratio of C18:2n-6 to C18:3n-3. Unpaired t-test or Mann-Whitney test were used in all analysis to compare CT and HF group. Spearman or Pearson test was used to analyze the correlations (* $p < 0.05$).

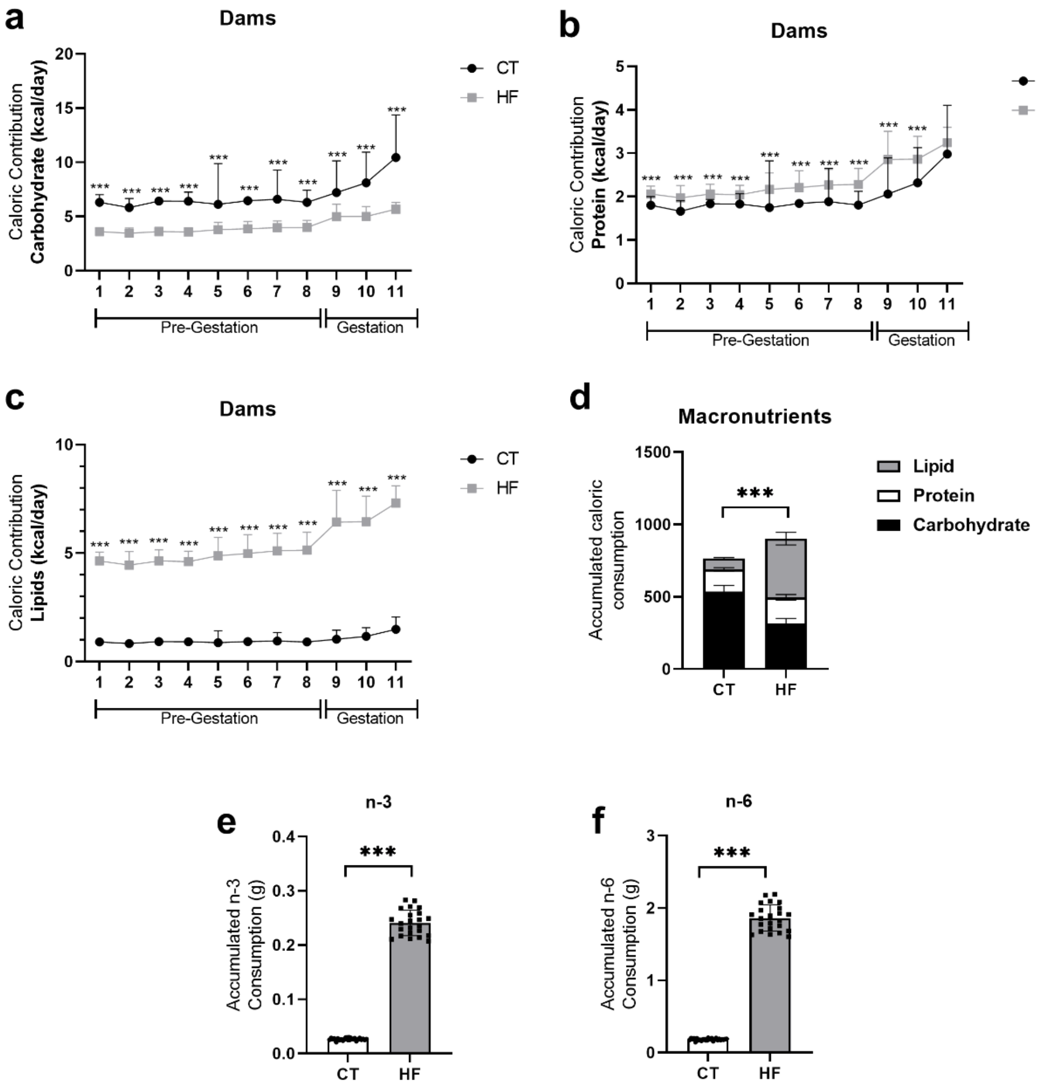

Figure S2. Caloric contribution of macronutrient intake of females fed high fat (HF) or control (CT) diet. (a) Caloric contribution of carbohydrates, (b) proteins and (c) lipids throughout the experimental period. Cumulative caloric contribution of (d) macronutrients, (e) omega-3 (n-3) and (f) omega-6 (n-6). Bars represent the mean ± standard deviation. Unpaired t-test, Mann-Whitney test or Two-way Anova were used in all analysis to compare CT and HF group at significance level (* $p < 0.05$,*** $p < 0.001$).

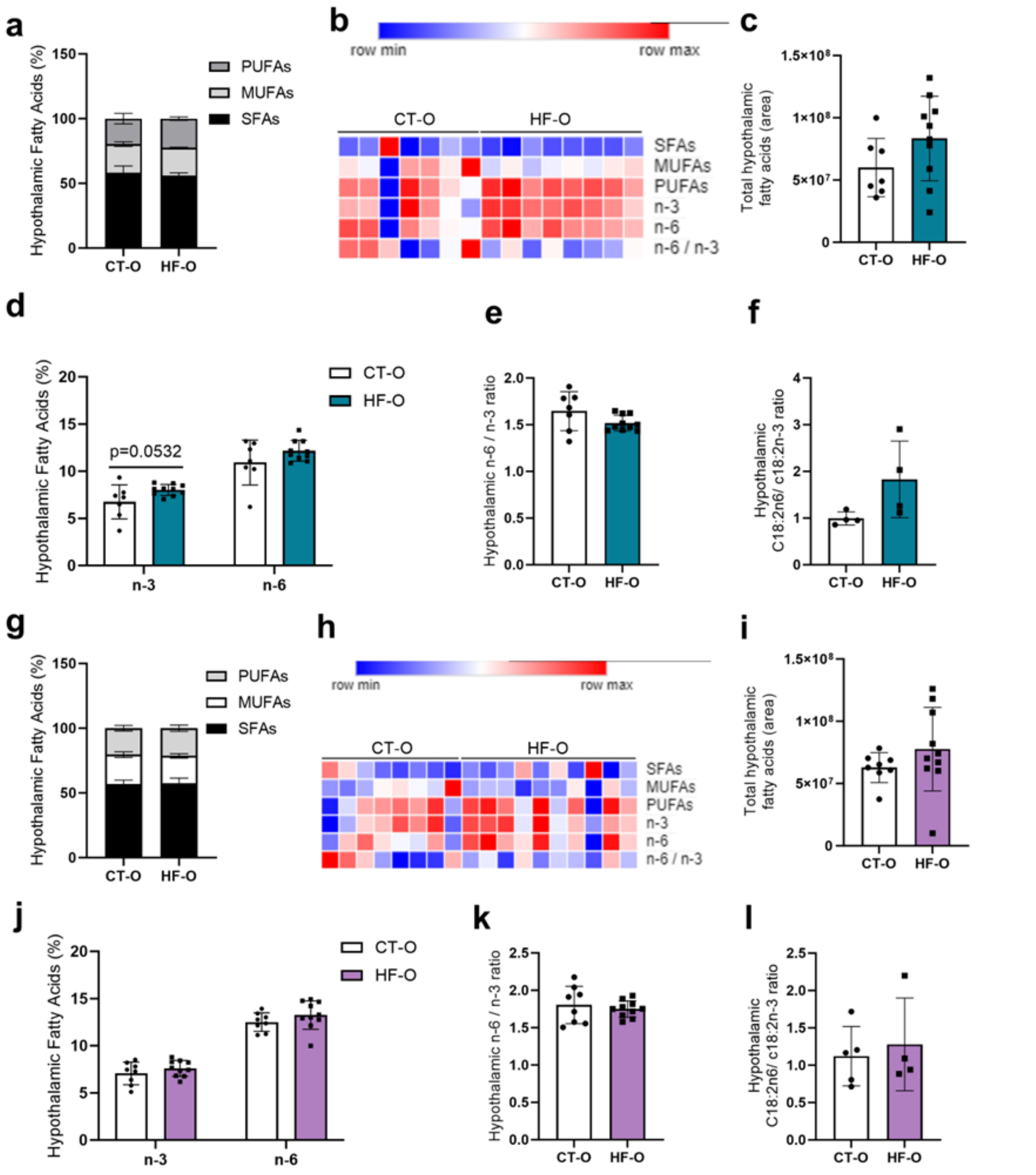

Figure S3. Fetal hypothalamic fatty acids profile. (a) Male hypothalamic Saturated Fatty Acids (SFAs), Monounsaturated Fatty Acids (MUFAs), Polyunsaturated Fatty Acids (PUFAs) proportion (CT-O n=7, HF-O n=10). (b) Male heat map by hypothalamic SFAs, MUFAs, PUFAs, ômega 3 (n-3) and ômega 6 (n-6) (CT-O n=7, HF-O n=10). (c) Male total hypothalamus fatty acids (area) (CT-O n=8, HF-O n=10). (d) Male hypothalamic proportion (%) of n-3 and n-6 (CT-O n=7, HF-O n=10). (e) Male n-6 to n-3 ratio in the hypothalamus (CT-O n=7, HF n=10). (f) Male hypothalamic ratio of C18:2n-6 to C18:3n-3 (CT-O n=4, HF-O n=4). (g) Female hypothalamic Saturated Fatty Acids (SFAs), Monounsaturated Fatty Acids (MUFAs), Polyunsaturated Fatty Acids (PUFAs) proportion (CT-O n=8, HF-O n=10). (h) Female heat map by hypothalamic SFAs, MUFAs, PUFAs, n-3 and n-6 (CT-O n=8, HF-O n=10). (i) Female total hypothalamus fatty acids (area) (CT-O n=8, HF-O n=10). (j) Female hypothalamic proportion (%) of n-3 and n-6 (CT-O n=8, HF-O n=10). (k) Female n-6 to n-3 ratio in the hypothalamus (CT-O n=8, HF-O n=10). (l) Female hypothalamic ratio of C18:2n-6 to C18:3n-3 (CT-O n=5, HF-O n=4). Spearman or Pearson test was used to analyze the correlations (* $p < 0.05$).
